# Going only half the way: cell cycle exit after the G1 restriction point

**DOI:** 10.1038/s41392-023-01692-1

**Published:** 2023-12-01

**Authors:** Lisa Müller, Tony Gutschner, Mechthild Hatzfeld

**Affiliations:** 1 Department of Pathobiochemistry, Institute of Molecular Medicine, Martin Luther University Halle-Wittenberg, Charles Tanford Protein Research Center, Halle, Germany; 2 Department of RNA Biology and Pathogenesis, Institute of Molecular Medicine, Martin Luther University Halle-Wittenberg, Charles Tanford Protein Research Center, Halle, Germany

**Keywords:** Cell biology, Molecular biology

**Fig. 1:**
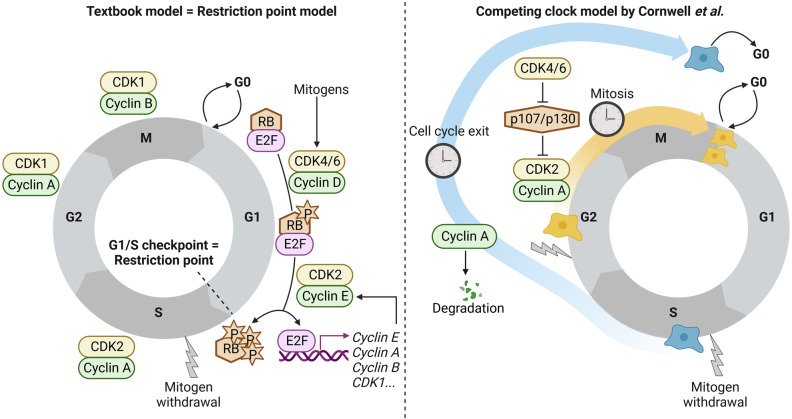
The textbook model of cell cycle progression is also known as the restriction point model (left panel) and proposes an irreversible commitment point at G1-S phase transition. Mitogens stimulate the cell to enter the cell cycle by promoting the assembly of cyclins with corresponding cyclin-dependent kinases (CDKs). Activated cyclin/CDK complexes phosphorylate the retinoblastoma protein (RB), which is released from transcription factors of the E2F family. Subsequently, E2Fs activate transcription, which allows cells to pass the restriction point. After passing the restriction point, a cell is committed to complete the round of cell cycle and becomes independent of mitogens. Different cyclin/CDK complexes control the transition of the cell through different cell cycle phases. Cornwell and colleagues developed a competing clock model (right panel) in which they proposed that mitogen withdrawal results in a decrease of cyclin A protein level resulting in cell cycle exit, even after passing the restriction point. Cells enter mitosis only if sufficient cyclin A protein is available to sustain CDK2 activity. Cyclin A transcription and CDK2 activity are regulated by CDK4/6 via the RB-family members p107/p130. Created with Biorender.com

In a recent article published in *Nature*, James A. Cornwell et al. describe that cyclin A2 levels determine whether cells will make an entrance into mitosis or whether cells will leave the cell cycle after having passed the G1/restriction (R)-point implicating that the decision is fully reversible (Fig. 1). This ground-breaking study could impact future approaches for cancer treatment by targeting the mitotic clock.^1^

The cell cycle includes several checkpoints to ensure the order, integrity, and fidelity of cell division. Growth factor activity decides whether cells pass the R-point and proceed from G1 into S-phase or whether cells withdraw from the cell cycle. According to the widely accepted “textbook model”, cells irreversibly commit to complete the cell cycle after having passed the R-point.^2^ During G1-phase, growth factors promote the assembly of D-type cyclins with the cyclin-dependent kinases (CDKs) CDK4 or CDK6 into functional complexes that phosphorylate proteins of the retinoblastoma (RB) family. Phosphorylation on multiple sites (“hyperphosphorylation”) leads to dissociation of RB from E2F thereby activating E2F-dependent transcription of target genes including cyclin E. Cyclin E in association with CDK2 supports RB hyperphosphorylation further thereby promoting the release of E2F and expression of cyclin E in a positive feedback loop. In addition, E2F transcription is stimulated in another positive feedback loop. This ensures that the level of cyclin E-CDK2 is sufficient to maintain RB hyperphosphorylation without the need of additional input from mitogens. As a consequence, cells are predicted to complete the cell cycle once they have passed the R-point.^3^ This mechanism precludes that cells reenter earlier stages of the cycle which could increase ploidy or interfere with chromosome segregation and is thus of fundamental importance for maintaining genome integrity.^4^ However, cell cycle exit after passing the R-point was observed in neural stem cells challenging this model.^5^

In an attempt to re-evaluate the R-point model, Cornwell et al. disrupted mitogen signaling in human cells by serum starvation or treatment with mitogen-activated protein kinase kinase (MEK) or CDK4/6 inhibitors. DNA content and RB phosphorylation analyses revealed that many S-phase cells completed DNA replication, maintained RB hyperphosphorylation and entered mitosis as expected. However, in contrast to the R-point model, a noteworthy number of “outlier cells” (up to 15%) proceeded to G2-phase but failed to complete mitosis. These cells were characterized by hypophosphorylated RB and 4 N DNA content. This suggests that these cells entered a “G0-like” state implicating a downregulation of the feedback loop between CDK2 and RB. This was confirmed in post R-cells by the gradual loss of CDK2 activity. Moreover, cells tend to leave the cell cycle when closer to the beginning of S-phase than to mitosis when growth factor signaling was blocked. Based on these observations, the authors propose a “competing clock model” that predicts the existence of two clocks in each cell: First, the mitosis clock, which measures the period from S- to mitosis phase. Second, the cell cycle exit clock, which measures the time needed to lose CDK2 activity after mitogen withdrawal. According to this model, cell fate depends on the balance between these two clocks: if the cell cycle exit clock is slower relative to the mitosis clock, mitosis is favored over cell cycle exit and vice versa. Therefore, blocking either clock would enable the opposing fate to win the rivalry. Indeed, deactivating the mitosis clock through inhibition of CDK1 combined with a block of mitogen signaling, either through growth factor removal, CDK4/6, or MEK inhibition, caused a loss of CDK2 activity in the majority of post-R cells which leaved the cell cycle. Importantly, this finding challenges the textbook model as it clearly shows that without CDK4/6 signaling, post-R cells are not able to maintain CDK2 activity.

If cell fate depends on endogenous clocks, what are the molecular timers of these clocks? In a series of experiments, the authors identify the stability of cyclin A2 as a key player in the cell cycle exit timing. Already a small reduction in cyclin A2 levels shortened the cell cycle exit clock timing, while the period from S-phase to mitosis was unchanged confirming that both clocks are driven by independent molecular processes.

Moreover, the experiments uncovered a role for CDK4/6 in regulating post-R cells: cyclin A2 transcription as well as CDK2 activity is affected, indicating an unexpected function of CDK4/6 in preserving CDK2 activity during S/G2-phase. In detail, CDK4/6 phosphorylated and inactivated the RB-related pocket proteins p107/p130, which allowed transcription of cyclin A2. Thus, cyclin A2 transcription was not regulated by a positive feedback loop between cyclin A2-CDK2 and E2Fs, but instead by the CDK4/6-dependent inhibition of negative regulators. Consequently, serum starvation or CDK4/6 inhibition maintained p107/p130 in an active state and allowed their association with inhibitory E2Fs (E2F4 and E2F5) to suppress cyclin A2 transcription resulting in low cyclin A2 protein as well as reduced CDK2 activity. To pass through G2-phase, cyclin A2-CDK2 activity is obligatory. Thus, cells with cyclin A2-CDK2 below a critical threshold will leave the cell cycle and enter a “G0-like” state, which is characterized by 4 N DNA content and hallmarks of senescence.

Cornwell and colleagues conclude that in most cells the R-point phenomenon is observed because cyclin A2 protein stability can maintain CDK2 activity after growth factor withdrawal which allows cells to enter mitosis. The competing clock model describes cell cycle exit versus mitosis as two mutually exclusive cellular fates, which are “timed” by the cyclin A2 protein level excluding a single molecular point where cells irreversibly commit to proliferation.

The elegant study by Cornwell and colleagues demonstrates the value of studying subpopulation of cells and raises some interesting questions concerning the molecular mechanisms regulating cyclin A protein stability as well as the temporal coupling of both clocks across different cell lines. Extending the mitosis clock using chemotherapy might synergize with CDK4/6 inhibition, but at the same time could increase toxicity. An important and still unanswered question refers to the fate of the 4 N cells that have exited the cell cycle. This could increase genomic instability and impact tumorigenesis thus warranting further investigations.
